# Mapping Paratope on Antithrombotic Antibody 6B4 to Epitope on Platelet Glycoprotein Ibalpha via Molecular Dynamic Simulations

**DOI:** 10.1371/journal.pone.0042263

**Published:** 2012-07-30

**Authors:** Xiang Fang, Ying Fang, Li Liu, Guangjian Liu, Jianhua Wu

**Affiliations:** Institute of Biomechanics/School of Bioscience and Bioengineering, South China University of Technology, Guangzhou, China; Consiglio Nazionale delle Ricerche, Italy

## Abstract

Binding of platelet receptor glycoprotein Ibα (GPIbα) to the A1 domain of von Willebrand factor (vWF) is a critical step in both physiologic hemostasis and pathologic thrombosis, for initiating platelet adhesion to subendothelium of blood vessels at sites of vascular injury. Gain-of-function mutations in GPIbα contribute to an abnormally high-affinity binding of platelets to vWF and can lead to thrombosis, an accurate complication causing heart attack and stroke. Of various antithrombotic monoclonal antibodies (mAbs) targeting human GPIbα, 6B4 is a potent one to inhibit the interaction between GPIbα and vWF-A1 under static and flow conditions. Mapping paratope to epitope with mutagenesis experiments, a traditional route in researches of these antithrombotic mAbs, is usually expensive and time-consuming. Here, we suggested a novel computational procedure, which combines with homology modeling, rigid body docking, free and steered molecular dynamics (MD) simulations, to identify key paratope residues on 6B4 and their partners on GPIbα, with hypothesis that the stable hydrogen bonds and salt bridges are the important linkers between paratope and epitope residues. Based on a best constructed model of 6B4 bound with GPIbα, the survival ratios and rupture times of all detected hydrogen bonds and salt bridges in binding site were examined via free and steered MD simulations and regarded as indices of thermal and mechanical stabilizations of the bonds, respectively. Five principal paratope residues with their partners were predicted with their high survival ratios and/or long rupture times of involved hydrogen bonds, or with their hydrogen bond stabilization indices ranked in top 5. Exciting, the present results were in good agreement with previous mutagenesis experiment data, meaning a wide application prospect of our novel computational procedure on researches of molecular of basis of ligand-receptor interactions, various antithrombotic mAbs and other antibodies as well as theoretically design of biomolecular drugs.

## Introduction

As a crucial step in a cascade of adhesion and signaling events in physiological hemostatic process, blood platelet adhesion to subendothelium of injured blood vessels is initiated by interaction of platelet glycoprotein Ibα (GPIbα) with its ligand von Willebrand factor (vWF) [1]. Under pathological conditions, this interaction can lead to thrombosis, an accurate complication causing heart attack and stroke [2]. Gain-of-function mutations in GPIbα (e.g. M239V) occur in patients with platelet-type von Willebrand disease (vWD) and then contribute to an abnormally high-affinity binding of platelets to vWF [3], [4]. The patients suffer from bleeding disorders, which are increased with ristocetin-induced platelet aggregation and characterized by intermittent thrombocytopenia and absent high molecular weight forms of plasma vWF [3], [4].

The resolved crystal structure of globular N-terminal domain of GPIbα is characterized by eight leucine-rich repeats (LRRs), a protruding flexible loop β-switch and a β-hairpin on the bottom [5]. The concave face of GPIbα binds A1 domain of vWF in a pincer-like grip with the β-switch and β-hairpin regions, in both the wild type and the mutant complex [6], [7]. Under high shear stress, flow may transform the β-switch from a flexible loop into a β-hairpin, and then enhance binding of vWF to GPIbα [8], [9], indicating a structural basis for GPIbα/vWF catch-bond such that increasing force on bond of GPIbα/vWF prolongs rather than shortens bond lifetimes [10]. Based on the significant role in thrombosis, GPIbα becomes a noteworthy target for antibodies and antithrombotic drugs. Many antibodies have been demonstrated to have various antithrombotic effects. Of known potent antibodies, antibody AK2, 24G10 and 6B4 can occupy completely the binding site of vWF-A1 domain on GPIbα [11], [12], whereas SZ-123 and SZ-125 compete with each other in targeting A3 domain of vWF [13].

The potent antithrombotic antibody 6B4, a murine monoclonal antibody (mAb) targeting the human GPIbα dose-dependently inhibits both the ristocetin- and botrocetin-induced binding of vWF to GPIbα, as well as human platelet adhesion to human collagen type I under flow [14]. 6B4 recognizes the epitope within the C-terminal flanking region (residues 201–268) of GPIbα to block binding of vWF to GPIbα [12], and injection of 6B4-Fab fragments has antithrombotic effect *in vivo* without prolongation of the bleeding time in Baboons [15]. With an iterative method of flexible docking alternating with mutagenesis experiments, five paratope residues of 6B4 have been identified to be Tyr^27D^, Lys^27E^, Asp^28^, Glu^93^ and Tyr^100C^ (Kabat numbering), which are located in the complementarity determining regions (CDRs) of light and heavy chain, respectively [16].

Mapping paratope to epitope with mutagenesis experiments in antithrombotic antibody researches is an essential topic but usually expensive, time-consuming and blind [16]. As an useful assistant, both rigid body and flexible docking program can be used to build various models of an antibody bound with an antigen to predict various residues involved in the interactions of the antibody and the antigen, but often fails to illustrate whether these residues are crucial or not for binding [16], possibly coming from that the conformation transforming is missed completely in rigid body docking or partly in flexible docking. A time-consuming experimental identification of these residues should be followed, as done in the work of Fontayne et al [16]. It is natural that, molecular dynamic (MD) simulation may be regarded an important tool in mapping paratope to epitope for antithrombotic antibody researches. By incorporating both conformational changes and atomic details of biomolecules in a 3D environment with different temperatures, pressures, and/or mechanical constraints, MD simulation can provide functional implication and yields information that is not possible through any other means [17], [18]. Variable simulation protocols and analysis methods such as free and steered MD have been developed for analyzing stability of a single molecule [19] or an isolated β-hairpin [9], hydrogen bond (H-bond) forming tendency between two contacting parts [9], [20], and unbinding of receptor from its ligand [10], [21].

Here, by combining with homology modeling, rigid body docking, free and steered MD simulation, we proposed a novel computational procedure to identify dominant residue pairs in interaction of paratope on 6B4 and epitope on GPIbα. Based on a best constructed model of 6B4 bound with GPIbα, we first examined thermal and mechanical stabilizations of bonds at binding site of 6B4/GPIbα complex via free and steered MD simulations, and then found that the stable hydrogen bonds and salt bridges, as the linkers between paratope and epitope residues, can be used in theoretically mapping of paratope to epitope. Our results were in good agreement with previous mutagenesis experiment data [16]. Our surprising results illustrated that the present computational procedure may find its application not only in the antithrombotic antibody researches but also in other biological topics, such as ligand-receptor interaction and computer-aided structure-based antibody drug design.

## Materials and Methods

**Figure 1:**
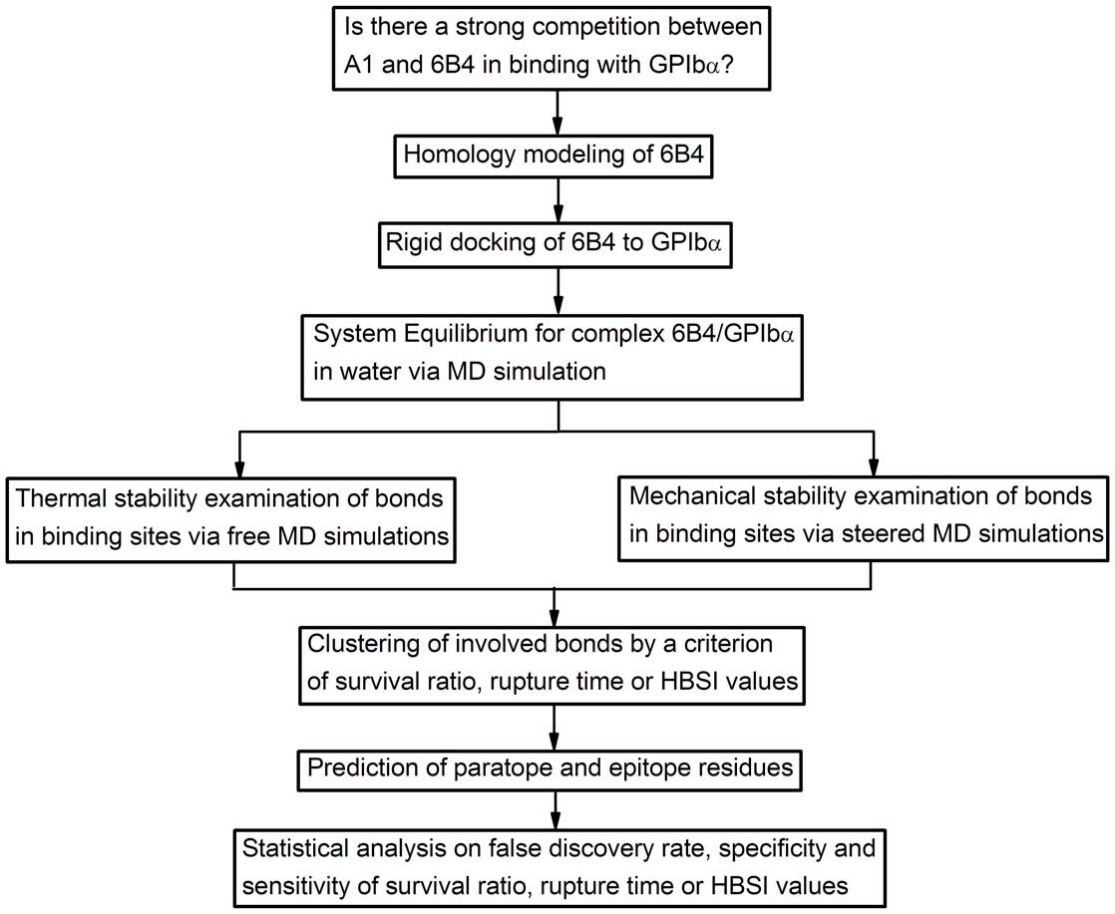
Ensemble workflow of computational procedure.

The strategy for theoretically mapping paratope on 6B4 to epitope on GPIbα was shown in the ensemble workflow of computational procedure (Fig. 1). All involved methods were described as below in detail.

### Homology modeling

The structure of 6B4 consists of light chain (V_L_ and C_L_ domains), heavy chain (V_H_ and C_H_ domains) and a 15-amino-acid (Gly_4_Ser)_3_ linker. We took amino acid sequence of 6B4 from a flexible docking model of 6B4/GPIbα [16] and obtained the templates of the sequence via PDB database search with BLAST [22] for homology modeling. The templates of (Gly_4_Ser)_3_ linker, heavy- and light- chains were the crystal structure of Fv antibody fragment (PDB code 1F3R), murine IgG1-Fab (PDB code 1GIG) and antibody 19G2 (PDB code 1UB5), respectively. To yield the most likely V_H_–V_L_ orientations, crystal structure of anti-sars ScFv antibody 80R (PDB code 2GHW) was used as a global template, based BLAST result that, among all antibody crystal structures containing the (Gly_4_Ser)_3_ linker in PDB, 2GHW structure has the highest sequence identity (48%) with 6B4-ScFv. The BLAST results revealed that the variable domain sequences of the light- and heavy- chain have, respectively, identities of 73 and 85% with their templates, the antibody 19G2 and the murine IgG1-Fab. Both the light- and heavy-chain sequences of their respective 120 and 113 residues were submitted to NCBI IgBlast (http://www.ncbi.nlm.nih.gov/igblast/) with IMGT (the international ImMunoGeneTics database) as Ig domain system [23], was then used to identify the six CDRs of 6B4. The paratope residues of 6B4 and locations of CDRs were summarized in Table 1, where the CDR H1, H2 and H3 in heavy chain were made up of their respective sequences, such as those from 26^th^ to 33^rd^ residue, from 51^st^ to 57^th^ residue and from 96^th^ to 110^th^ residue, whereas the sequences from 162^nd^ to 172^nd^ residue, from 190^th^ to 192^nd^ residue and from 229^th^ to 237^th^ residue 26 contributed to the CDR L1, L2 and L3 in heavy chain, respectively. An alignment between 6B4 and its templates was generated by ClustalX [24], then homology modeling of 6B4 structure were performed by Modeller 9v6 [25]. Eight 6B4-Fv models of so-called single chain Fv (ScFv), which was arranged in V_H_–V_L_ orientation and joined together with (Gly_4_Ser)_3_ linker [26], [27], [28], were built up. Of these models, one with a small Z-score value of −1.181 was regarded as a native-like model and selected for further docking to GPIbα.

**Table 1:** The CDRs and identified paratope residues of 6B4 [16].

Paratope residue	Serial number*	Location of CDR	
		Name	Location in sequence†
Tyr	106	CDR H1	26–33
Tyr	166	CDR H2	51–57
Lys	167	CDR H3	96–110
Asp	168	CDR L1	162–172
Glu	233	CDR L2	190–192
		CDR L3	229–237

* The positions of residues in 6B4-ScFv were expressed with serial numbering from N-terminal of heavy chain to C-terminal of light chain.

† The serial numbers, 166, 167, 168, 233 and 106, are corresponding to those in Kabat numbering, such as 27D, 27E, 28, 93 and 100C, respectively. ^†^The residue sequences, contributed to their respective CDRs (CDR H1, H2, H3, L1, L2 and L3), follow the serial numbering too.

### Docking

The ligand-free GPIbα (PDB code 1M0Z) was regarded as the receptor with ligand 6B4. Docking of 6B4 to GPIbα (amino acids 1–266) was performed with ZDOCK3.0 [29]. As indicated in previous works [12], we also designated the β-switch region of GPIbα as the binding site of 6B4 CDRs. In docking to a fixed GPIbα, 6B4 was translated and/or rotated with 6° sampling density in rotational space. With rigid body docking, 54,000 poses were generated. All complexes were analyzed and scored by Zrank [30]. Of these 54,000 poses, only the top 20 complexes ranking with negative Z-rank score were taken [31] for visual inspection with VMD [32], and the 339^th^ complex with the lowest Z-rank score of −73.4 was regarded as the best or most possible native-like model, which was used in MD simulations. The best 6B4/GPIbα model was analyzed with the software PSAIA (Protein Structure and Interaction Analyzer), a powerful verification tool in docking [33], and Van Der Waals, hydrophobic and polar interactions were obtained by the newly developed algorithm PIADA (Protein Interaction Atom Distance Algorithm).

### MD simulations

Two software packages, visual molecular dynamics (VMD) for visualization and modeling [32], and NAMD 2.6 program for free and steered MD simulations [34], were used in our simulations. 6B4/GPIbα complex, the 339^th^ complex of 54,000 poses generated by Zdock was solvated with TIP3P water molecules in a rectangular box of 16.5 nm×9.3 nm×7.1 nm. The system was neutralized by adding 93 Na^+^ and 92 Cl^−^ ions. The CHARMM22 all-atom protein force field [35], along with CMAP correction for backbone, particle mesh Ewald algorithm for electrostatic interaction and a 1.3 nm cutoff for electrostatic and van der Waals interaction, were used to perform simulations with time step of 2 fs and periodical boundary condition.

The system equilibrium was performed twice, along the protocol as that, firstly at 0° K, the system was subjected to an energy minimization of 1,000 time steps with heavy or non-hydrogen protein atoms being fixed, another energy minimization of 5,000 time steps with all atoms free ensues; Then, the system temperature was raised from 0° K to 310° K in 20 ps, and the system was further equilibrated for 5 ns with temperature and pressure control. The temperature was held at 310° K using Langevin dynamics, and the pressure was held at 1 atm by the Langevin piston method. The time-curves of the temperature, total energy and RMSD of heavy atoms were used to observe whether the system had been equilibrated or not after passing time of 5 ns (Figure S1 in Suppl. Materials). Two equilibrated complex structures were obtained from two corresponding equilibrated systems described above, and taken as the two initial conformations for free and steered MD simulations.

### Free and steered MD simulations

Free MD simulations were run thrice on each of equilibrated systems over 3 ns with time step 2 fs and disabled control of temperature and pressure. The interactions of residues in 6B4 CDRs with those in GPIbα were explored by VMD. The salt bridges and/or H-bonds, which might contribute to the binding of 6B4 to GPIbα, were detected by a bond-length cutoff of 0.35 nm and bonding angle of 30 degree, beyond which the bonds were considered to be disrupted, but only the bond-length cutoff was applied to examine the salt bridges in binding site. The survival probability of a H-bond and/or salt bridge were approximately evaluated by the ratio of bond survival time in the period of simulation.

Steered MD simulations also were run thrice on each of two equilibrated systems with the C-terminal C_α_ atoms, of both heavy and light chains of 6B4, being fixed and N-terminal C_α_ atom, of GPIbα, being steered. Along the direction vertical to the line between two C-terminal C_α_ atoms of heavy and light chains of 6B4, the pulling over 6 ns was performed with time step 2 fs and a constant velocity of 1 nm/ns, at which the pulling would contribute to H-bond rupture with conservation of secondary structures of 6B4 [36] (Movie S1 in Supp. Materials). The virtual spring, connecting the dummy atom and the steered atom, had a spring constant of 7000 pN/nm. Three stretching events were simulated for each of two different initial equilibrated structures. The rupture times of different hydrogen bonds under stretching were recorded to examine the mechanical strengths or stabilizations of H-bonds.

### Stabilization index and clustering of hydrogen bond

Besides survival ratio and rupture time, we here introduced another H-bond stabilization index (HBSI) to score the stabilization of a hydrogen bond under both stretching and thermal excitation, with definition as that HBSI_j_ = (ω_j_+α_j_)/2, where HBSI_j_ expresses HBSI value of the j^th^ H-bond, ω_j_ = max{ω_j1_, ω_j2_}, α_j_ = θ_j_/max{θ_1_, θ_2_,…, θ_N_}, θ_j_ = max{θ_j1_, θ_j2_}, N is the total number of involved H-bonds, ω_j1_ and θ_j1_ are the mean survival ratio and rupture time of j^th^ H-bond detected, respectively, from thrice free and steered MD simulations with the i^th^ initial equilibrated complex conformation, for j = 1, 2, …, N, and i = 1, 2.

Such definition of HBSI make 0≤HBSI≤1. Generally, both the mean survival ratio ω_j1_ and mean rupture time θ_j1_ are initial-state dependent, namely, θ_j1_≠θ_j2_ and ω_j1_≠ω_j2_. The normalized mean rupture time α_j_ expresses the relative mechanical strength of the j^th^ H-bond, and together with mean survival ratio ω_j_, contributes to score importance of the bond in intermolecular interaction between paratope and epitope. HBSI_j_, the H-bond stabilization index of the j^th^ H-bond, synthesizes the effects of both thermal stabilization and mechanical strength of the bond on the paratope-epitope interactions.

We defined that a bond is low, moderate and high stable, if anyone of its mean survival ratio, normalized mean rupture time and HBSI index lies in region from 0 to 0.3, from 0.3 to 0.55 and from 0.55 to 1.0, respectively. A high stable bond observed from both free and steered MD simulation may have either or both of thermal and mechanical high stabilization, based on the differences between its mean survival ratio, normalized mean rupture time and HBSI index. All observed bonds were clustered into three groups, the low, moderate and high stable one, with above definition of bond stabilization. Statistical analyses were performed with Student's t test for different bond groups.

### Evaluation of false discovery rate and the sensitivity and specificity of stabilization indices of hydrogen bond

In examining the false discovery rate (FDR) and the sensitivity and specificity of three stabilization indices (such as the mean survival ratio, normalized mean rupture time and HBSI), a residue was expected to be positive for its contribution at least on one bond with high stabilization, and negative if it was involved just in formation of low and moderate stable bond(s). In bonds contributed by a residue, only the maximum stabilization index was used to appraise whether the residue was either a positive or a negative one. A positive residue was assumed to be either a paratope or epitope one, and a negative one did not. The mutagenesis experiment data [16] were used to determine whether a positive residue is false or true. Denoting the numbers of the true and false positive residues by TP and FP, the false discovery rate (FDR) could be evaluated by


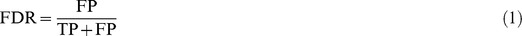


Required in determination of the sensitivity and specificity, the numbers of true and false negative residues were usually unknowns for the rare mutagenesis experiment data. Here, we further define a random process X _j_ so that, at any time in duration of observation, X_j_ takes value 1 if one bond forms between the j^th^ predicted negative residue and its partner in binding site and zero otherwise, for j = 0, 1, 2, …, M, where M is the number of uncertain negative residues; and these random processes of M are independent each other, in other words, the formation and breakage of a bond is not related to other bonds. Thus, E(X_j_ ), the expected value of X_j_, is evaluated here by the maximum of either the mean survival ratios, or the normalized mean rupture times or HBSI values of involved bonds. Denoting the possible number of false negative residues in all uncertain negative residues by FN_1_, we have


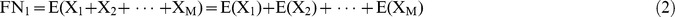


Thus, the sensitivity and specificity could be evaluated by


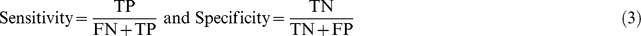


Where, TN and FN are the numbers of all true and false negative predicted- residues, respectively, TN = TN_0_+(M−FN_1_), FN = FN_0_+FN_1_, and TN_0_ and FN_0_ express the true and false negative identified-residue numbers, respectively.

## Results

### Less information of paratope residues and their partners are provided by Docking results

**Figure 2:**
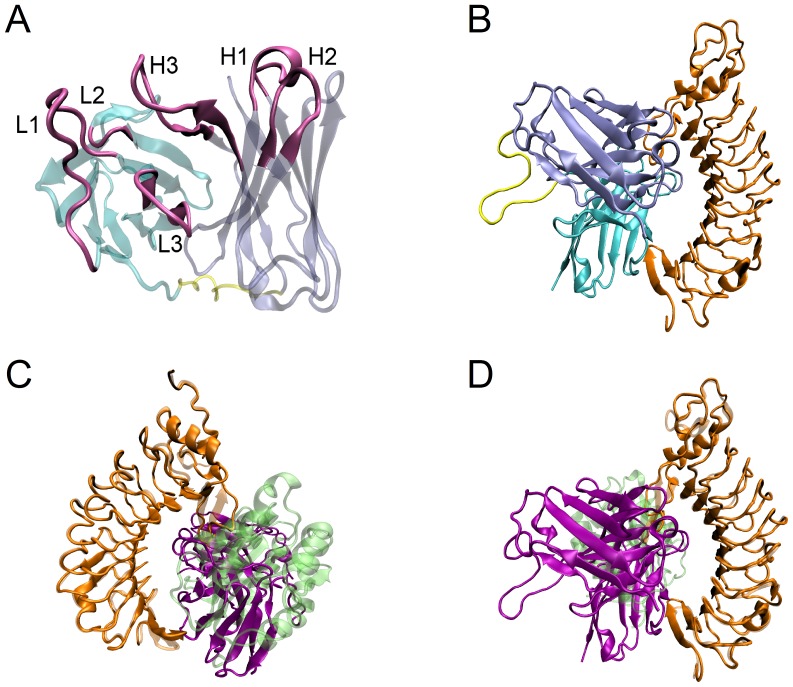
Models of free and bound 6B4. A, model of 6B4-ScFv via Homology modeling, where the heavy chain (iceblue), light chain (cyan) and (Gly_4_Ser)_3_ linker (yellow) were shown in transparent newcartoon representation, and the six complementarity determining regions (CDRs) (mauve), i.e. CDR H1, H2, H3, L1, L2 and L3, were marked; B, conformation of the 339^th^ complex of 6B4 bound to GPIbα subunit (orange); C, structural superposition of 6B4/GPIbα and A1-GPIbα complex (PDB code 1SQ0), where A1 is shown in transparent lime and 6B4 in prunosus; D, the back side view of C.

Through homology modeling, we built a model of 6B4-ScFv (Fig. 2 A), in which six CDRs, distributed on the top of both light- and heavy- chains, will contribute to binding of 6B4 to GPIbα. This 6B4 model may be native-like for its low Z-score value of −1.181, and the high identities, 73% and 85%, of the light- and heavy- chains with their respective templates, the antibody 19G2 and the murine IgG1-Fab (see Material and Methods). The 339^th^ complex (Fig. 2 B), a 6B4/GPIbα model with the lowest Z-rank score of −73.4, was picked out from 54,000 poses generated by docking of above 6B4-ScFv to ligand-free GPIbα (see Material and Methods). This complex model may be the best one, because the lowest Z-rank score means its conformation is energy favorable [30].

The structural superposition between 6B4/GPIbα and wild-type complex of GPIbα-A1 (PDB code 1SQ0) (Fig. 2 C and D) also expressed a competitive binding of 6B4 and vWF to GPIbα, or say, 6B4 does prevent vWF-A1 being bound to GPIbα, regardless of being induced by ristocetin, botrocetin or shear stress [14]. With PSAIA software [33], we found that, 29 residues on GPIbα were involved in 57 interactions (including polar, hydrophobic and Van der Waals interactions) of vWF-A1 and GPIbα. Among these 29 residues in GPIbα, 18 residues were occupied by those of bound 6B4 in the 339^th^ complex, saying that 6B4 could occupy about 62% ( = 18/29) of the binding site of A1. So, the 6B4/GPIbα model (Fig. 2 B) might be biologically meaningful, because binding site of vWF-A1 on GPIbα is mostly occupied by 6B4, which prevents A1 from interacting with GPIbα due to the obvious steric hindrance (Fig. 2 C and D) [14].

We analyzed the best 6B4/GPIbα model with the software PSAIA [33], and obtained 79 Van Der Waals, 6 hydrophobic and 5 polar interactions with the newly developed algorithm PIADA. Regardless of Van Der Waals interactions, we obtained seven residues, namely, Met^102^, Leu^231^, Val^232^, Thr^101^, Arg^194^, Tyr^234^ and Glu^233^ on 6B4, which might be involved in hydrophobic and polar interaction with corresponding residues on GPIbα (Table 2), but only Glu^233^ was pre-determined (Table 1) [16]. This result showed that, deriving from docking analysis of one complex pose, less information on intermolecular interaction at binding site of 6B4/GPIbα would fail in theoretically mapping of paratope residues to their partners, even if this pose conformation is energy favorable. The reason may come from that, firstly, the best 6B4/GPIbα model was not a equilibrated structure if it is surrounded with water molecules under physiological thermal environment; secondly, the docking analysis for the hydrophobic and polar interaction of paratope with epitope via PSAIA was based on a static complex pose, whereas this complex in water would undergo a random conformational transition.

**Table 2:** Hydrophobic and polar interactions from docking analysis.

No	Hydrophobic residue pairs		Polar residue pairs	
	6B4	GPIbα	6B4	GPIbα
1	Met^102^	Leu^178^	Thr^101^	Lys^231^
2	Met^102^	VAL^236^	Arg^194^	Glu^14^
3	Leu^231^	Val^234^	Val^232^	Asp^235^
4	Val^232^	Val^234^	Tyr^234^	Ala^238^
5	Val^232^	Val^236^	Glu^233^	Asp^235^
6	Val^232^	Ala^238^		

### Paratope residues are involved in hydrogen bonding for the equilibrated complex

**Figure 3:**
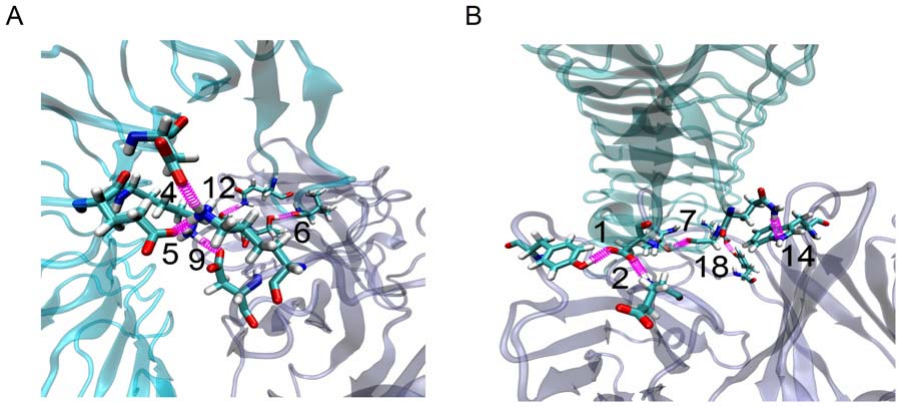
Conformation of the 339^th^ complex after the first (A) and second (B) equilibration. GPIbα (cyan) and 6B4 (iceblue) are shown in transparent newcartoon representation. All ten bonds were numbered with the index listed in Table 3. The 5^th^, 4^th^ and 9^th^ bonds express the three salt bridges, others are H-bonds.

We obtained two equilibrated 6B4/GPIbα complex structures (Fig. 3 A and B) by performing a system equilibrium twice, along a same protocol of energy minimization and with the assumption that, the system would be equilibrated after passing time of 5 ns (Materials and Methods), because the time-curves of the temperature, total energy and RMSD of heavy atoms were fluctuated on their respective stable levels with small relative derivations (Figure S1 in Suppl. Materials). To verify the hypothesis of that H-bonds on binding site can identify key residue pairs in paratope and epitope, we obtained five H-bonds (Table 3), which were involved in the intermolecular interactions of paratope and epitope and explored by VMD [32], for each of the initial equilibrated conformations.

**Table 3:** Summary of survival ratios, rupture times and involved residues of hydrogen bonds and salt bridges obtained from free and steered simulations.

Bond		GPIbα		6B4		ECC*		Survival Ratio		Rupture time (ns)	
No	Type	Residue	Atom	Residue	Atom	I	II	I	II	I	II
1	H	Asp^235^	OD1	Tyr^166^	OH		+		0.94±0.01		3.37±0.45
2	H	Asp^235^	OD2	Glu^233^	N		+	0.38±0.14	0.72±0.12	1.78±0.54	2.46±0.06
3	S	Asp^235^	OD2	Lys^167^	NZ						0.17±0.16
4	S	Asp^175^	OD2	Lys^167^	NZ	+		0.49±0.23		3.07±1.43	0.18±0.24
5	S	Glu^151^	OE2	Lys^167^	NZ	+		0.08±0.09		0.54±0.30	
6	H	Val^234^	O	Thr^101^	OG1	+		0.35±0.08		1.46±0.61	
7	H	Gly^233^	O	Thr^101^	OG1		+	0.46±0.06	0.51±0.315	1.64±0.61	1.74±0.04
8	H	Gly^233^	O	Thr^101^	N				0.33±0.39		0.44±0.03
9	S	Lys^152^	NZ	Asp^168^	OD2	+		0.88±0.09		4.49±0.39	
10	H	Gln^232^	O	Tyr^234^	OH			0.53±0.03	0.47±0.33	1.26±0.66	1.41±0.31
11	H	Gln^232^	OE1	Ser^100^	OG			0.03±0.03			
12	H	Gln^232^	NE2	Ser^100^	OG	+		0.20±0.13		0.83±0.36	0.10±0.15
13	H	Gln^232^	NE2	Thr^53^	OG1				0.05±0.01		
14	H	Gln^232^	NE2	Trp^52^	NE1		+				
15	H	Gln^232^	NE2	Arg^31^	O			0.03±0.02			
16	H	Asn^110^	O	Arg^31^	NH2						0.05±0.03
17	S	Glu^181^	OE2	Arg^31^	NH1			0.31±0.23			
18	H	Glu^40^	OE1	Tyr^110^	OH		+		0.20±0.15		0.52±0.40
19	H	Glu^40^	OE2	Tyr^110^	OH				0.18±0.04		0.63±0.42
20	S	Glu^40^	OE2	Arg^97^	NH1				0.15±0.07		1.85±0.24
21	H	Lys^237^	NZ	Leu^164^	O			0.03±0.03			
22	H	Lys^237^	NZ	Ser^163^	O			0.06±0.01		0.28±0.30	
23	H	His^86^	ND1	Tyr^32^	OH				0.20±0.09		0.76±0.34

* ECC is an abbreviation of equilibrated complex conformation. Letter H and S expresses the two types of bonds, such as hydrogen bond and salt bridge, respectively. The heading I and II denote two different ECCs of of 6B4 bound to GPIbα, the superscript numbers on residues (Column 5 and 3) designate the positions of their respective involved residues in sequences of 6B4 and GPIbα with serial numbering, and, the donor- and acceptor-atoms (Column 6) on paratope residues (Column 5) together with their respectively partners (Column 4) on epitope residues (column 3) contribute to bonds in binding site. All bonds, which were derived from static analyses and/or from thrice independent free and steered MD simulations with equilibrated conformation I and II, respectively, were designated by the symbol “+” and/or nonzero values (mean ± SD) of survival ratios and rupture times of bonds.

Our results (Table 3) showed that, of the ten detected H-bonds, the 5^th^ and 4^th^ bonds would be contributed to two salt bridges between doner residue Lys^167^ on CDR L1 and its two partners, Glu^151^ and Asp^175^ on GPIbα, and the 9^th^ bond was involved in another salt bridge from Asp^168^ on CDR L1 to Lys^152^ on GPIbα (Fig. 3 A); similar to Lys^167^, the doner residue Thr^101^ on CDR H3 also had two acceptor residues, Val^234^ and Gly^233^ on GPIbα, to form the 6^th^ (Fig. 3 B) and 7^th^ H-bond (Fig. 3 A), respectively; with a same doner residue Gln^232^ on GPIbα, both Ser^100^ on CDR H3 and Trp^52^ on CDR H2 were respectively related to the 12^th^ and 14^th^ bond (Fig. 3 A and B), and also with a same acceptor residue Asp^235^ on GPIbα, the 1^st^ and 2^nd^ bond were formed by Tyr^166^ on CDR L1 and Glu^233^ on CDR L3 (Fig. 3 B), respectively; in the 18^th^ bond (Fig. 3 B), the doner residue Tyr^110^ on CDR H3 was paired with its acceptor residue Glu^40^ on GPIbα.

From above results, we obtained that, besides Glu^233^ shown in docking analysis, other three identified paratope residues, Tyr^166^, Lys^167^ and Asp^168^ and Glu^233^ were also emerged from above ten residue pairs (Table 1 and 3) [16], which were detected just from two equilibrated conformations. Even so, in recognizing the key paratope residues with the corresponding H-bonds, less knowledge on behaviors of these detected hydrogen bonds made us to be confused by the fragmentary and superabundant H-bond messages.

### Paratope and epitope can be mapped by the involved H-bonds of high survival rates

With use of bond-length cutoff of 0.35 nm and bonding angle of 30 degree (see Materials and Methods), we examined the events of breaking and forming of bonds by performing free MD simulations thrice on each of initial equilibrated conformation I and II of 6B4/GPIbα (Fig. 3 A and B) for 3 ns, and found that, of all possible H-bonds and/or salt bridges (Table 3), two salt bridges and ten H-bonds were newly generated and others were pre-observed in two different initial poses. All bonds (Table 3) from free MD simulation could be clustered into three groups of low, moderate and high thermal stabilization, or the instantaneous, unstable and stable groups, by their corresponding survival ratio values ranging from 0 to 0.3, from 0.3 to 0.55 and from 0.55 to 1.0, respectively (Material and Methods). The instantaneous contained the 5^th^, 11^th^–13^th^, 15^th^ and 18^th^–23^rd^ bonds, the unstable group included the, 4^th^, 6^th^–8^th^, 10^th^ and 17^th^ bonds, and the stable group was consisted of the 1^st^, 2^nd^ and 9^th^ bonds. The survival ratio values (mean ± SD) of the high, moderate and low stable groups were 0.84±0.12, 0.44±0.23 and 0.13±0.12, respectively. Statistical analyses with Student's t test showed significant differences in the three groups (*p*<0.01).

**Figure 4:**
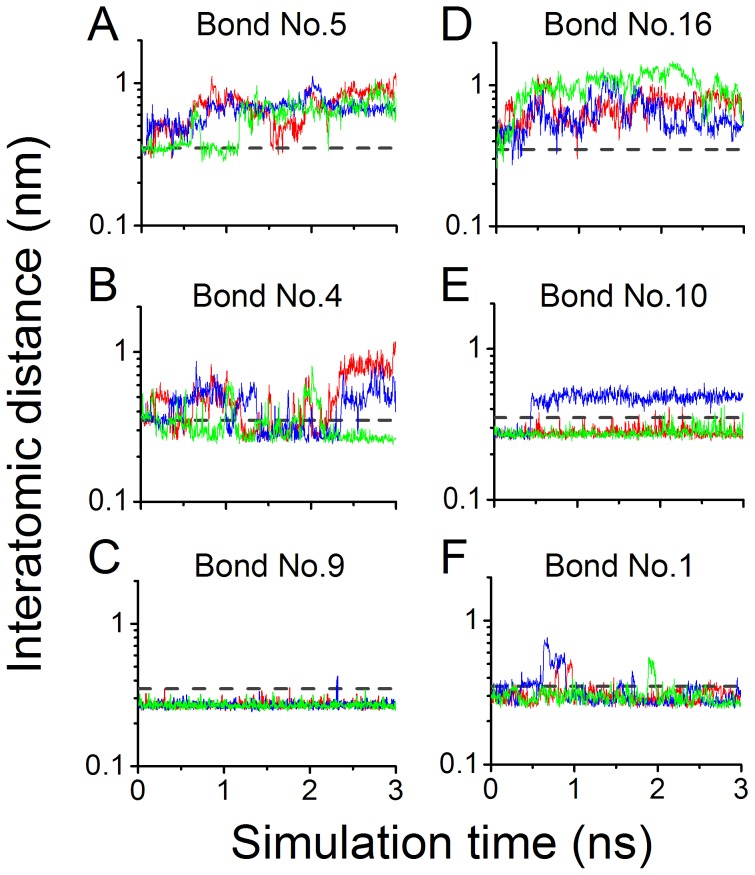
Time courses of interatomic distances of six representative bonds in binding site of 6B4/GPIbα complex. The interatomic distances of six representative bonds were plotted against simulation time, where the interatomic distances were from the oxygen atoms of acidic residues and their respective partners, the nitrogen atoms of basic residues, for three salt bridges, 5^th^ (A), 4^th^ (B) and 9^th^ (C) bonds, or from doners to their respective acceptors for three hydrogen bonds, 16^th^ (D), 10^th^ (E) and 1^st^ (F) bonds. The salt bridges and hydrogen bonds were simulated with the initial conformation I (Fig. 3 A) and II (Fig. 3 B), respectively. The gray dashed line expresses the distance cut-off of 0.35 nm beyond which the bonds breaks, and the blue, green and red lines exhibit the variation of interatomic distances (nm) of a bond against simulation time (ns) for thrice-repeat independent free MD simulations, respectively. The thermal stabilizations of the 4^th^ and 10^th^ bonds (B and E) seemed to be higher than those of the 5^th^ and 16^th^ bonds but lower than those of the 9^th^ and 1^st^ bonds. Remarkable difference in the thrice-repeat independent simulations showed a random behavior of intermolecular interactions.

We found from *D-t* curves (Fig. 4), the time courses of distance between two bonding atoms, that the three representative H-bond pairs, namely, the 16^th^ and 5^th^ bonds, the 10^th^ and 4^th^ bonds as well as the 1^st^ and 9^th^ bonds, showed to be instantaneous, unstable and stable one with low, moderate and high survival rates, respectively. The instantaneous H-bonds, the 16^th^ and 5^th^ bonds, whose *D-t* curves (Fig. 4 A and D) were both in fluctuating above their cutoff line, broke readily in most of the simulation period, showing little constraints in motion of the bonding atom pairs, such as Ser^100OG^ and Gln^232OE1^, and Trp^52NE1^ and Gln^232NE2^; on the contrary, the 9^th^ bond, a salt bridge between Asp^168OD2^ and Lys^152NZ^, was stable, and almost remained intact for its *D-t* curve (Fig. 4 C) almost always being below its cutoff line during the simulations, and so do the 1^st^ bond, implying that the corresponding donor Tyr^166OH^ was in close contact with its acceptor Asp^235OD1^ (Fig. 4 F); and for two unstable H-bonds, the 4^th^ bond, a salt bridge between Lys^167NZ^ and Asp^175OD2^, and 10^th^ bond between Gln^232O^ and Tyr^234OH^, their corresponding *D-t* curves (Fig. 4 B and E) both had an irregular fluctuation around their respective cutoff lines, suggesting moderate survival probabilities of the two bonds over the simulation time of 3 ns.

The results (Table 3) showed that, the mean survival ratios of these detected H-bonds varied from zero to 0.94, showing the marvelous complexity of paratope-epitope interaction which was referred closely to not only the number but also the behaviors of involved salt bridges and/or H-bonds. For the 1^st^, 9^th^, 2^nd^, 10^th^, 7^th^ and 4^th^ bonds, their respective mean survival ratios of 0.94, 0.88, 0.72, 0.53, 0.51 and 0.49 were in top 6 among those of all detected H-bonds from thrice free MD simulations with both equilibrated conformation I and II; the paratope residue Glu^233^, Lys^167^ and Asp^168^, together with their respective epitope residue Asp^235^, Asp^175^ and Lys^152^, contributed to three salt bridges, the 2^nd^, 4^th^ and 9^th^ bonds; and, the paratope residue Tyr^166^, Thr^101^ and Tyr^234^ were paired with Asp^235^, Gly^233^ and Gln^232^ to form the 1^st^, 7^th^ and 10^th^ H-bonds. In above six paratope residues involved in H-bonds with mean survival ratios in top 6, the residue Glu^233^, Tyr^166^, Lys^167^ and Asp^168^ are pre-determined through mutagenesis experiments [16], meaning that the paratope and epitope residues may be mapped by the involved H-bonds of moderate and high thermal stabilizations; and the two unidentified residues, Thr^101^ and Tyr^234^, may play important roles in 6B4/GPIbα interactions too. Perhaps, the residue pairs, which were involved in H-bonds with low mean survival ratios (small than 0.35) beyond top 6, were needed to pay special attentions in paratope and epitope mapping, for their smaller contributions on paratope-epitope interactions than those with mean survival ratios in top 6. Yet, the 14^th^ H-bond obtained in static state analysis might be very weak, because it was missed in free MD simulations. It means that, a weak H-bond, such as 14^th^ H-bond, derived from static state analysis on one pose of complex in water, might remain very short time and almost have no contribution to binding of 6B4 to GPIbα.

### H-bonds with high mechanical stabilization contribute to mapping of paratope and epitope

As a linker between paratope and epitope, an H-bond or salt bridge will regulate binding of 6B4 to GPIbα. Similar to the thermal stabilization reflected by the survival rates, the mechanical strengths or stabilizations of H-bonds may impact remarkably the intermolecular interaction of paratope and epitope. To test this hypothesis, we performed steered MD simulation thrice on each of the equilibrated conformation I and II over 6 ns with pulling velocity of 1 nm/ns, and along unbinding pathway (as termed by Isralewitz et al [37]) of 6B4/GPIbα complex under stretching (Figure S2, Movie S1 in Suppl. Materials), recorded the distances in each bonding atom pairs one by one to evaluate the rupture times of involved H-bonds.

**Figure 5:**
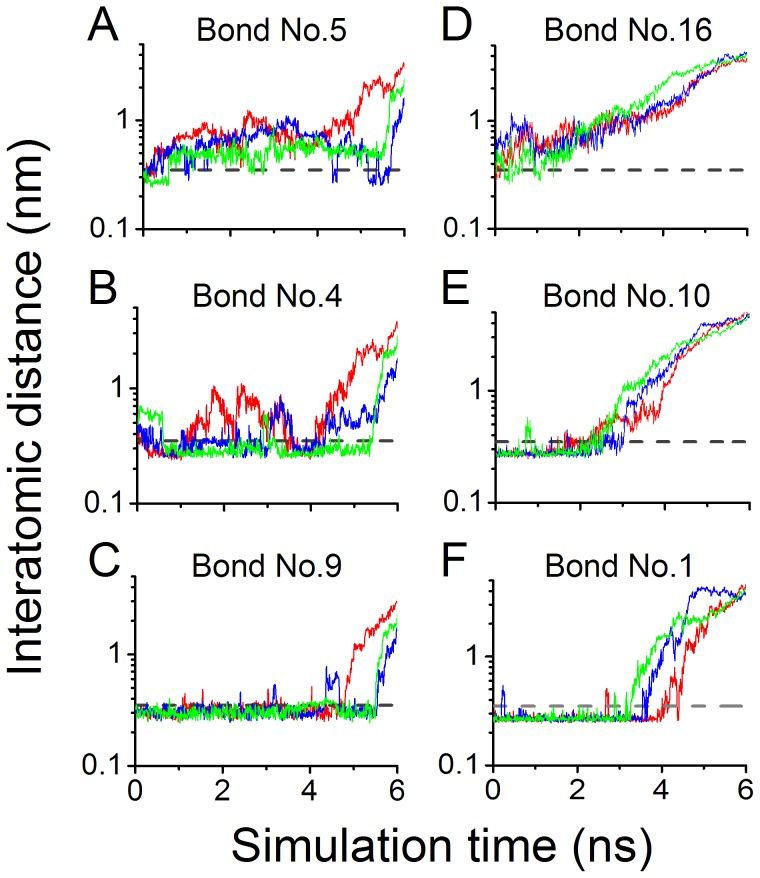
Variation of interatomic distance versus steered simulation time. The interatomic distances of the six representative bonds under stretching were plotted against simulation time, where all descriptions for line types, bonds and their lengths are same as in Figure 4. These time courses of interatomic distances showed that, the 5^th^ and 16^th^ bonds were very quickly ruptured (A and D), in comparison with others, in which the 9^th^ and 1^st^ bonds would maintain more long time (C and F) than 4^th^ and 10^th^ bonds (B and E).

All results were summarized in Table 3, from which we found that, the 14^th^ H-bond vanished also in steered MD simulation as shown in free MD simulation; besides those observed from one pose of complex (Fig. 3 A and B), eight new events of hydrogen bonding in binding sites occurred at binding site; and all detected bonds under stretching had their respective different mean rupture times in range from zero almost to 4.49 ns. Figure 5 showed the *D*-*t* curves for representative H-bonds, such as the 16^th^ and 5^th^ bonds, the 10^th^ and 4^th^ bonds as well as the 1^st^ and 9^th^ bonds. In pulling, the distances between each bonding atom pairs would across over the cutoff of 0.35 nm quickly for the 16^th^ and 5^th^ H-bonds (Fig. 5 A and D), maintain few nano seconds and then steeply increase over the cutoff for the 4^th^, 10^th^, 9^th^ and 1^st^ H-bonds (Fig. 5 B, E, C and F), showing the weak, moderate or strong mechanical constraints between these bonding atom pairs, respectively.

Obviously, the longer the rupture time of an H-bond under stretching, the stronger the mechanical strength of the bond, and the higher the mechanical stabilization of the bond. All bonds observed from SMD simulations were also partitioned into three bond types of weak, moderate and strong mechanical stabilization by the normalized mean rupture times ranging from 0 to 0.3, 0.3 to 0.55 and 0.55 to 1.0, respectively (Materials and Methods). Of these three bond groups, one with strong mechanical stabilization contained the 9^th^, 1^st^ and 4^th^ bonds, the second with moderate mechanical stabilization included the 2^nd^, 20^th^, 7^th^, 6^th^ and 10^th^ bonds, and the third with weak mechanical stabilization was consisted of the 3^rd^, 5^th^, 8^th^, 12^th^, 16^th^, 18^th^, 19^th^, 22^nd^ and 23^rd^ bonds. The normalized rupture time values (mean ± SD) of the strong, moderate and weak bond types were 0.81±0.22, 0.40±0.12 and 0.10±0.10, respectively. Differences between the three groups were significant (*p*<0.01).

In comparison with free MD simulation, the H-bonds in the top 6 of mean survival ratios are in good accordance with those in the top 6 of mean rupture times (Figure S3), indicating a positive association between thermal and mechanical stabilization for each bonding atom pairs, regardless of the 10^th^ and 20^th^ bond. Astonishing, as a results, the pre-determined paratope residue Glu^233^, Tyr^166^, Lys^167^ and Asp^168^ (Table 1 and 3) [16] were involved also in H-bonds in top 4 of mean rupture times (Table 3), suggesting that H-bonds with high mechanical stabilizations may be used to recognize key paratope and epitope residues, similar to results from free MD simulation.

### H-bond stabilization index reveals importance of involve residues in paratope-epitope interaction

**Figure 6:**
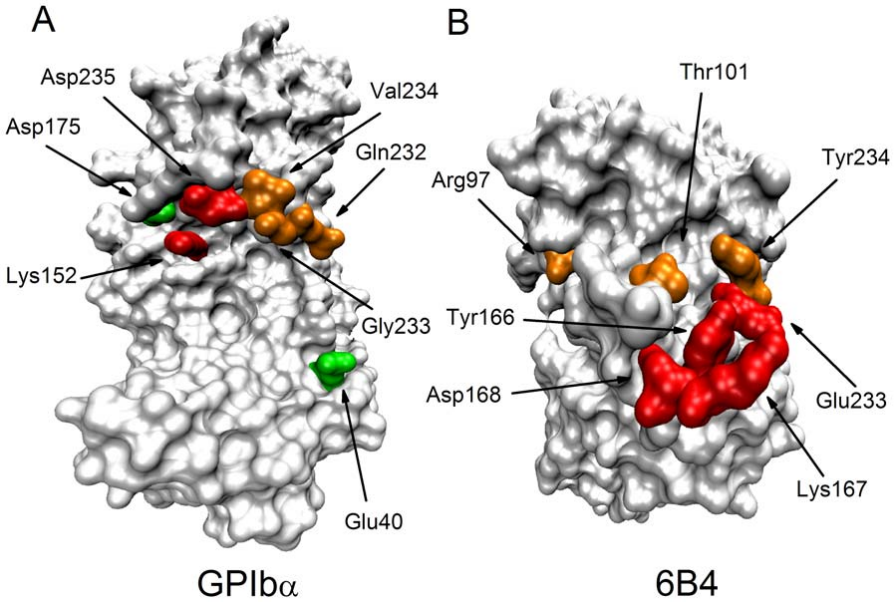
Residues involved in H-bonds and salt bridges of top 8 HBSI values. Red, significantly disrupted binding when mutated; orange, mutagenesis data are unavailable; green, no obvious effect was observed when mutated. A, surf representation of GPIbα; B, surf representation of 6B4.

We here further used a hydrogen bond stabilization index (HBSI) (Materials and Methods), normalized in a range scale from 0 to 1 and synthesized both thermal and mechanical stabilizations of H-bonds in binding sites, as a score in mapping paratope to epitope. The values of HBSI were evaluated with the survival ratios and rupture times of bonds derived from MD simulations (Table 3), and then also used to clustered all observed bonds in three groups with low, moderate and high stabilization, by HBSI values ranging from 0 to 0.3, 0.3 to 0.55 and 0.55 to 1.0, respectively (Materials and Methods). The paratope residues and their partners (Fig. 6) with HBSI values in top 8 were listed in Table 4, which showed such a gradually weakened importance of involved residue pairs on intermolecular interactions between paratope and epitope that the values of HBSI decreased from 0.94 to 0.28 along the pathway from the 9^th^, 1^st^, 2^nd^, 4^th^, 7^th^, 10^th^, 6^th^ to the 20^th^ bond, a feature different not only from survival ratios but also from rupture times.

**Table 4:** Hydrogen bonds and salt bridges with higher stabilization in Top 8.

Rank	Bond No.	HBSI*	Interaction residue pairs	
			GPIbα	6B4
1	9	0.94	LYS^152^	ASP^168^
2	1	0.85	ASP^235^	TYR^166^
3	2	0.64	ASP^235^	GLU^233^
4	4	0.59	ASP^175^	LYS^167^
5	7	0.45	GLY^233^	THR^101^
6	10	0.42	GLN^232^	TYR^234^
7	6	0.34	VAL^234^	THR^101^
8	20	0.28	GLU^40^	ARG^97^

* HBSI expresses the index of hydrogen bond stabilization (Materials and Methods).

Maybe, in mapping paratope to epitope, a bond with a larger HBSI value is more important than that with a lower HBSI value, because the larger the HBSI value, the higher the thermal and/or mechanical stabilization. Interestingly, the 9^th^, 1^st^, 2^nd^ and 4^th^ bonds, in which the pre-identified paratope residue Tyr^166^, Lys^167^, Asp^168^ and Glu^233^ [16] arose, were in top 4 in rankings of HBSI. This result was similar to that from rupture times, and in comparison, only the residue Lys^167^ would lose in bonds in top 4 of survival ratios. Even so, of the stabilization indices in mapping paratope to epitope, HBSI should be regarded as a better one than those of the survival ratio and the rupture time, because it contained information of the thermal and mechanical stabilizations of the bonds.

Of the five residue pairs with top 5 of HBSI values, the epitope residue Asp^235^ on GPIbα is also identified by mutagenesis experiments [16], same as paratope residue Tyr^166^, Lys^167^, Asp^168^ and Glu^233^ on 6B4; mutating Gly^233^ to Val^233^, a known gain-of-function mutant, would stabilize the β-hairpin conformation, and let to a 5- to 6-fold reduced affinity for binding of 6B4 to mutant GPIbα, meaning Gly^233^ to be structurally essential [12]; the interaction between 6B4 and GPIbα was impaired notably by mutating Lys^152^ to Ala [16], although the importance of Lys^152^ had not been completely confirmed, because of the possible conformational change induced by the mutation. Our results proposed Thr^101^ on 6B4 and Asp^175^ on GPIbα as a possible key residue pairs, in spite of less knowledge on their contribution to binding of 6B4 to GPIbα; and, out of our expectation, although the importance of Lys^167^ had been confirmed, mutating its partner Asp^175^ did not markedly impair the binding [16], possibly coming from that this mutation might enhance the interactions of Lys^167^ to its two other partners GLU^151^ and Asp^235^ on GPIbα (Table 3).

### False discovery rate, sensitivity and specificity of H-bond stabilization index

To test whether the HBSI index is better than other two stabilization indices (the mean survival ratio and normalized mean rupture time) or not in mapping paratope to epitope, we evaluated the false discovery rate (FDR), the sensitivity and specificity for each of the three stabilization indices by assigning all involved residues to two clusters, the positive and negative one, with a positive criterion score of 0.55 (see Materials and Methods; Table S1, S2 and S3 in Suppl. Materials). The differences between the two groups for each of the three stabilization indices were significant (*p*<0.01). All results were shown in Table 5.

**Table 5:** The false discovery rate, sensitivity and specificity of three different positive criterions.

Positive criterion	False discovery rate	Sensitivity	Specificity
Mean survival ratio	0.00	0.48	1.00
Normalized mean rupture time	0.17	0.53	0.92
HBSI	0.14	0.61	0.94

The false discovery rate was evaluated by Eq. 1, and Eq. 3 was used to estimated the sensitivity and specificity, with use of data in Table S2 in Suppl. Materials.

We found that, derived from the HBSI criterion, seven positive residues were predicted to be Asp^168^, Lys^167^, Tyr^166^ and Glu^233^ on 6B4 as well as Asp^235^, Lys^152^ and Asp^175^ on GPIbα (Table S1); of above seven positive residues, the first five were true positive for their mutagenesis experiment data (Table 1) [16], Lys^152^ was also regarded as a true positive one because mutating Lys^152^ to Ala impaired notably the interaction of 6B4 and GPIbα [16], but Asp^175^ was false positive for the less effect of mutating Asp^175^ to Ala on binding [16]; with criterion of the normalized mean rupture time, all above seven residues except Glu^233^ were also clustered in a positive group, and Glu^233^ became a false negative one; and in comparison, only Asp^168^, Tyr^166^, Glu^233^, Asp^235^ and Lys^152^ were classified to positive group, but Lys^167^ and Asp^175^ were the false and true negative ones, respectively, based on the mean survival ratio criterion. The possible numbers of false negative residues in all uncertain negative residues were expected to be 4.4, 3.4 and 3.9 (see Materials and methods, Table S1 and S2), according to the mean survival ratio, normalized mean rupture time and HBSI indices, respectively.

Based above results, the false discovery rates of the mean survival ratio, the normalized mean rupture time and HBSI indices were evaluated to be 0.00, 0.17 and 0.14 (Table 5), respectively. It indicated that, in comparison with HBSI index criterion, decreasing of positive residues led to a lower level of FDR for the mean survival ratio criterion, and did not so for the normalized mean rupture time criterion. However, a FDR level of 0.14 should be acceptable in identifying the paratope and epitope residues, so that HBSI index criterion might be a better one than others for its ability to predict true positive residues as much as possible. The sensitivities of the mean survival ratio normalized mean rupture time and HBSI indices were estimated as 0.48, 0.53 and 0.61, respectively. Less difference was in specificities (1.0, 0.92 and 0.94) of the mean survival ratio, the normalized mean rupture time and HBSI indices. These results showed that, in comparison with mean survival ratio and normalized mean rupture, HBSI index is better in mapping paratope to epitope for its higher sensitivity with an acceptable FDR and almost without losing of its specificity.

## Discussion

Identification of key residues involved in binding sites is an essential topic in antibody research. A conventional route to determine paratope- and/or epitope-residues was along with an alternating process of antibody mutagenesis experiment and docking analysis, and might be time-consuming and ineffective, possibly mainly coming from that the static interaction analysis, which provided insufficient messages on both paratope and epitope so that antibody mutagenesis experiments would be blind [12], [16]. A novel computational procedure, combining with homology modeling, rigid body docking, free and steered molecular dynamic simulations, was proposed here to identify the paratope residues on 6B4 and their partners on GPIbα. Our results showed that most identified paratope residues can be predicted via the present procedure, with hypothesis of that these residues would involved in stable H-bonds between paratope and epitope.

Our results illustrated that a 6B4 model (Fig. 2 A) constructed through homology modeling seemed native-like for its better mapping of paratope on 6B4 to epitope on GPIbα (Fig. 2 B and C). Distinct from the iterative approach via docking and mutating [16], we herein proposed rigid body docking rather than flexible docking [38], because the least of known experimental data was required. From docking analysis, we just found Glu^233^ on 6B4, one of five identified paratope residues [16], suggesting that docking analysis cannot provide enough messages on key paratope residues and their partners. By contrast, of ten H-bonds detected in binding sites for two poses of equilibrated 6B4/GPIbα (Fig. 3 A and B), four identified paratope residues emerged, indicating that some paratope residues were involved in hydrogen bond interactions more closely than in both Van Der Waals and hydrophobic interactions. Even so, the superabundant and fragmentary messages of H-bonds detected from the two conformation were not enough available in determining whether the doner-acceptor residue pairs are the key residue pairs of paratope and epitope or not.

Physically, the atoms of 6B4/GPIbα in liquid or physiological environment will fluctuate irregularly around their equilibrium positions, and the conformational transformation from one to another ensues, accompanying with intermolecular interaction in paratope and epitope. Being correlated closely with Van Der Waals and hydrophobic as well as polar interaction, breaking and reforming of H-bonds in binding site would be the dominant events in binding of ligand to receptor. These H-bonds with high survival possibilities may be the determinants in mapping paratope to epitope. Thereby, to gain a profound insight on these H-bonds, we further investigated their thermal and mechanical stabilizations by performed free and steered MD simulations, which had been used for investigating conformational stability [19], behaviors of H-bonds [9], [20], and residue interactions in unbinding of receptor from its ligand under stretching [10], [21].

However, we found that the conformational evolution of 6B4/GPIbα under stretching and thermal excitation might be initial-state dependent, namely, the conformational transition from one to another is not only dependent on both the stretching and thermal excitation but also on initial conformation. This initial-state dependence was reflected by the involved H-bonds in 6B4/GPIbα interface, as shown in Table 3, which indicated that the numbers, survival ratios and rupture times of the involved H-bonds as well as the types of the doner-acceptor residue pairs would vary with initial conformation of 6B4/GPIbα. It suggested us to perform each of free and steered MD simulations with two or more different equilibrated structures for detecting all possible paratope- and epitope-residues. And, not only a free but also a steered MD simulation just modeled a thermal response process of 6B4/GPIbα with a given equilibrated structure in water, so we here had performed each of free and steered MD simulations thrice at least to obtain an approximate result with statistical significance. The random feature and the initial-state dependence of conformational evolution might lead to fail in detecting paratope residue Tyr^100C^ (Table 1), and possibly, performing enough simulations in parallel are beneficial in locating this residue.

Surely, our results indicated that H-bonds with high values of survival ratios or rupture times can provide a clue in recognizing key paratope residues and their partners. However, we were confused with whether the 6^th^, 7^th^, 10^th^ or the 20^th^ H-bond had more importance on intermolecular interaction, because of their moderate values of survival ratios and rupture times. Rationally, H-bonds with high mechanical and/or thermal stabilization will form a tight constraint to their respective doner-acceptor residue pairs under stretching and thermal excitation. So, we here suggested a hydrogen bond stabilization index (HBSI), which are reflected synthetically by both survival ratio and rupture time, as a score in mapping paratope to epitope. Based on rankings of HBSI values, we found that the 7^th^ bond, as a linker among paratope and epitope, is stronger than the 6^th^, 10^th^ and 20^th^ bonds. For Gly^233^ and Thr^101^, the residue pair involved in the 7^th^ H-bond, it was indicated that Gly^233^ is important to maintain not only stabilization of the β-hairpin conformation but also affinity for binding of 6B4 to mutant GPIbα [12], in spite of less knowledge on contribution of Thr^101^ to 6B4 in binding to GPIbα. And, in other residue pairs with HBSI values in top 4, paratope residue Tyr^166^, Lys^167^, Asp^168^ and Glu^233^ were identified by mutagenesis experiments [16]. It means that HBSI, as an index in recognizing key epitope residues and their partners, is more suitable than the survival ratio or the rupture time. In fact, HBSI characterized evenly both the thermal and mechanical stabilizations of bonds involved in interactions of paratope- and epitope- residues. As a result, both the false discovery rate and the specificity, being respectively equal to 0.14 and 0.94, of HBSI index were located at a moderate level in comparison with those of other two indices (Table 5). This compromise on the false discovery rate and the specificity made HBSI index not only having higher sensitivity than those of other two indices but also being a better one than others for its ability to predict both paratope- and epitope residues as much as possible, with a acceptable FDR level of 0.14 and without loss of its specificity.

The validation process exposed herein justified that the HBSI index may be a suitable measure not only in identifying but also in further defining a reliable H-bond network, which reflects interactions of paratope- and epitope- residues in binding site. Nevertheless, the present work, which was restricted to the adoption of just one complex (6B4/GPIbα) studied plus the availability of few verified interactions of paratope- and epitope- residues, is a first step in computationally mapping paratope to epitope. For general forecasting considerations about the formation of antibody/antigen complexes, the HBSI index still need an extensible validation improvement from more case studies of other similar documented antibodies, such as AK2 and 24G10 [11], [12], [16]. However, our results showed that, the present computational procedure should make antibody research to be less time-consuming, because the key paratope- or epitope-residues predicted via MD simulation will provide a useful clue to mutation experiments, especially for the antibodies without both crystal structures and mutation experiment data. Besides, this method may be used to reveal the molecular mechanism underlying the reciprocal competitive binding of the antibodies, such as AK2, 24G10 and 6B4, to GPIbα [11], [12], [16], and regulating the physiological and pathological processes of thrombosis and Haemophilia [2], [4], and find its application on researches of other various antibodies, molecular basis of ligand-receptor interactions, and theoretically design of biomolecular drugs.

## Supporting Information

Figure S1 **Variation of the temperature, total energy and RMSD of heavy atoms of 6B4/GPIbα complex against simulation time.** (A) and (B) express the time-courses of the temperature, total energy and RMSD of heavy atoms of 6B4/GPIbα complex in two independent system equilibrium processes, respectively. The time step is 2 fs.
(TIF)

Figure S2 **Variation of force on complex under stretching against simulation time.** (A) and (B) are the force profiles with two different initial equilibrated complex conformations, respectively, at pulling velocity of 1 nm/ns. The time step is 2 fs, and the data are means of three independent unbinding events.
(TIF)

Figure S3 **Correlation between normalized rupture time and survival ratio.**
(TIF)

Movie S1 **Unbinding of 6B4/GPIbα complex simulated by steered MD simulation followed the first system equilibration.** The complex is shown in newcartoon representation. The heavy chain, light chain and (Gly_4_Ser)_3_ linker of 6B4 are indicated with iceblue, cyan and yellow, respectively; The GPIbα subunit is shown in orange. The fixed atoms (Cα atoms of 6B4 heavy chain C-terminal residue Ser120 and light chain C-terminal residue Arg248) are indicated as green spheres; the steered atom (Cα atom of GPIbα C-terminal residue Thr266) is shown as a red sphere.
(MPG)

Table S1 **Summary of the identified positive and negative residues on 6B4 and GPIbα.**
(DOC)

Table S2 **Unidentified negative residues and their false negative probabilities.**
(DOC)

Table S3 **The numbers of positive and negative residues derived from three different positive criterions.**
(DOC)

## References

[1] Savage B, Saldivar E, Ruggeri ZM (1996) Initiation of platelet adhesion by arrest onto fibrinogen or translocation on von Willebrand factor. Cell 84: 289–297. 8565074 10.1016/s0092-8674(00)80983-6

[2] Ruggeri ZM (2002) Platelets in atherothrombosis. Nat Med 8: 1227–1234. 12411949 10.1038/nm1102-1227

[3] Russell SD, Roth GJ (1993) Pseudo-von Willebrand disease: a mutation in the platelet glycoprotein Ib alpha gene associated with a hyperactive surface receptor. Blood 81: 1787–1791. 8384898

[4] Sadler JE (2005) New concepts in von Willebrand disease. Annu Rev Med 56: 173–191. 15660508 10.1146/annurev.med.56.082103.104713

[5] Huizinga EG, Tsuji S, Romijn RA, Schiphorst ME, de Groot PG, et al (2002) Structures of glycoprotein Ibalpha and its complex with von Willebrand factor A1 domain. Science 297: 1176–1179. 12183630 10.1126/science.107355

[6] Sadler JE (2002) Biomedicine. Contact–how platelets touch von Willebrand factor. Science 297: 1128–1129. 12183613 10.1126/science.1075452

[7] Dumas JJ, Kumar R, McDonagh T, Sullivan F, Stahl ML, et al (2004) Crystal structure of the wild-type von Willebrand factor A1-glycoprotein Ibalpha complex reveals conformation differences with a complex bearing von Willebrand disease mutations. J Biol Chem 279: 23327–23334. 15039442 10.1074/jbc.M401659200

[8] Chen Z, Lou J, Zhu C, Schulten K (2008) Flow-induced structural transition in the beta-switch region of glycoprotein Ib. Biophys J 95: 1303–1313. 18441028 10.1529/biophysj.108.132324 PMC2479615

[9] Lou J, Zhu C (2008) Flow induces loop-to-beta-hairpin transition on the beta-switch of platelet glycoprotein Ib alpha. Proc Natl Acad Sci U S A 105: 13847–13852. 18772372 10.1073/pnas.0801965105 PMC2544542

[10] Yago T, Lou J, Wu T, Yang J, Miner JJ, et al (2008) Platelet glycoprotein Ibalpha forms catch bonds with human WT vWF but not with type 2B von Willebrand disease vWF. J Clin Invest 118: 3195–3207. 18725999 10.1172/JCI35754 PMC2518822

[11] Shen Y, Romo GM, Dong JF, Schade A, McIntire LV, et al (2000) Requirement of leucine-rich repeats of glycoprotein (GP) Ibalpha for shear-dependent and static binding of von Willebrand factor to the platelet membrane GP Ib-IX-V complex. Blood 95: 903–910. 10648402

[12] Cauwenberghs N, Vanhoorelbeke K, Vauterin S, Westra DF, Romo G, et al (2001) Epitope mapping of inhibitory antibodies against platelet glycoprotein Ibalpha reveals interaction between the leucine-rich repeat N-terminal and C-terminal flanking domains of glycoprotein Ibalpha. Blood 98: 652–660. 11468163 10.1182/blood.v98.3.652

[13] Zhao Y, Dong N, Shen F, Xie L, He Y, et al (2007) Two novel monoclonal antibodies to VWFA3 inhibit VWF-collagen and VWF-platelet interactions. J Thromb Haemost 5: 1963–1970. 17723136 10.1111/j.1538-7836.2007.02682.x

[14] Cauwenberghs N, Meiring M, Vauterin S, van Wyk V, Lamprecht S, et al (2000) Antithrombotic effect of platelet glycoprotein Ib-blocking monoclonal antibody Fab fragments in nonhuman primates. Arterioscler Thromb Vasc Biol 20: 1347–1353. 10807753 10.1161/01.atv.20.5.1347

[15] Wu D, Meiring M, Kotze HF, Deckmyn H, Cauwenberghs N (2002) Inhibition of platelet glycoprotein Ib, glycoprotein IIb/IIIa, or both by monoclonal antibodies prevents arterial thrombosis in baboons. Arterioscler Thromb Vasc Biol 22: 323–328. 11834536 10.1161/hq0202.102321

[16] Fontayne A, De Maeyer B, De Maeyer M, Yamashita M, Matsushita T, et al (2007) Paratope and epitope mapping of the antithrombotic antibody 6B4 in complex with platelet glycoprotein Ibalpha. J Biol Chem 282: 23517–23524. 17569666 10.1074/jbc.M701826200

[17] Adcock SA, McCammon JA (2006) Molecular dynamics: Survey of methods for simulating the activity of proteins. Chemical Reviews 106: 1589–1615. 16683746 10.1021/cr040426m PMC2547409

[18] Long M, Sato M, Lim CT, Wu JH, Adachi T, et al (2011) Advances in Experiments and Modeling in Micro- and Nano-Biomechanics: A Mini Review. Cellular and Molecular Bioengineering 4: 327–339.

[19] Weaver DF, Sutherland JJ, O'Brien LA, Lillicrap D (2004) Molecular modeling of the von Willebrand factor A2 domain and the effects of associated type 2A von Willebrand disease mutations. Journal of Molecular Modeling 10: 259–270. 15322948 10.1007/s00894-004-0194-9

[20] Huang QS, Lou JZ, Wu JH, Zhu C (2011) Conformational Transition of Glycoprotein Ib alpha Mutants in Flow Molecular Dynamics Simulation. Cellular and Molecular Bioengineering 4: 495–504.

[21] Interlandi G, Thomas W (2010) The catch bond mechanism between von Willebrand factor and platelet surface receptors investigated by molecular dynamics simulations. Proteins 78: 2506–2522. 20602356 10.1002/prot.22759 PMC6107302

[22] Altschul SF, Madden TL, Schaffer AA, Zhang J, Zhang Z, et al (1997) Gapped BLAST and PSI-BLAST: a new generation of protein database search programs. Nucleic Acids Res 25: 3389–3402. 9254694 10.1093/nar/25.17.3389 PMC146917

[23] Lefranc MP, Pommie C, Ruiz M, Giudicelli V, Foulquier E, et al (2003) IMGT unique numbering for immunoglobulin and T cell receptor variable domains and Ig superfamily V-like domains. Dev Comp Immunol 27: 55–77. 12477501 10.1016/s0145-305x(02)00039-3

[24] Thompson JD, Gibson TJ, Plewniak F, Jeanmougin F, Higgins DG (1997) The CLUSTAL_X windows interface: flexible strategies for multiple sequence alignment aided by quality analysis tools. Nucleic Acids Res 25: 4876–4882. 9396791 10.1093/nar/25.24.4876 PMC147148

[25] Sali A, Blundell TL (1993) Comparative protein modelling by satisfaction of spatial restraints. J Mol Biol 234: 779–815. 8254673 10.1006/jmbi.1993.1626

[26] Dai K, Zhu H, Ruan C (2003) Generation and characterization of recombinant single chain Fv antibody that recognizes platelet glycoprotein Ibalpha. Thromb Res 109: 137–144. 12706643 10.1016/s0049-3848(03)00152-x

[27] Arcangeli C, Cantale C, Galeffi P, Rosato V (2008) Structure and dynamics of the anti-AMCV scFv(F8): effects of selected mutations on the antigen combining site. J Struct Biol 164: 119–133. 18662789 10.1016/j.jsb.2008.06.013

[28] Wilkinson IC, Hall CJ, Veverka V, Shi JY, Muskett FW, et al (2009) High resolution NMR-based model for the structure of a scFv-IL-1beta complex: potential for NMR as a key tool in therapeutic antibody design and development. J Biol Chem 284: 31928–31935. 19776018 10.1074/jbc.M109.025304 PMC2797264

[29] Mintseris J, Pierce B, Wiehe K, Anderson R, Chen R, et al (2007) Integrating statistical pair potentials into protein complex prediction. Proteins 69: 511–520. 17623839 10.1002/prot.21502

[30] Pierce B, Weng Z (2007) ZRANK: reranking protein docking predictions with an optimized energy function. Proteins 67: 1078–1086. 17373710 10.1002/prot.21373

[31] Pierce B, Weng Z (2008) A combination of rescoring and refinement significantly improves protein docking performance. Proteins 72: 270–279. 18214977 10.1002/prot.21920 PMC2696687

[32] Humphrey W, Dalke A, Schulten K (1996) VMD: visual molecular dynamics. J Mol Graph 14: 33–38, 27–38. 8744570 10.1016/0263-7855(96)00018-5

[33] Mihel J, Sikic M, Tomic S, Jeren B, Vlahovicek K (2008) PSAIA - protein structure and interaction analyzer. BMC Struct Biol 8: 21. 18400099 10.1186/1472-6807-8-21 PMC2364630

[34] Phillips JC, Braun R, Wang W, Gumbart J, Tajkhorshid E, et al (2005) Scalable molecular dynamics with NAMD. J Comput Chem 26: 1781–1802. 16222654 10.1002/jcc.20289 PMC2486339

[35] MacKerell AD, Bashford D, Bellott M, Dunbrack RL, Evanseck JD, et al (1998) All-atom empirical potential for molecular modeling and dynamics studies of proteins. Journal of Physical Chemistry B 102: 3586–3616. 10.1021/jp973084f 24889800

[36] Zhang B, Su ZC, Tay TE, Tan VB (2010) Mechanism of CDK5 activation revealed by steered molecular dynamics simulations and energy calculations. Journal of Molecular Modeling 16: 1159–1168. 20013135 10.1007/s00894-009-0629-4

[37] Isralewitz B, Baudry J, Gullingsrud J, Kosztin D, Schulten K (2001) Steered molecular dynamics investigations of protein function. J Mol Graph Model 19: 13–25. 11381523 10.1016/s1093-3263(00)00133-9

[38] Dominguez C, Boelens R, Bonvin AM (2003) HADDOCK: a protein-protein docking approach based on biochemical or biophysical information. J Am Chem Soc 125: 1731–1737. 12580598 10.1021/ja026939x

