# Supplementary figures and images for: Mapping Paratope on Antithrombotic Antibody 6B4 to Epitope on Platelet Glycoprotein Ibalpha via Molecular Dynamic Simulations
Source: PLoS One. 2012 Jul 30;7(7):e42263. doi: 10.1371/journal.pone.0042263 (PMC3408434; doi:10.1371/journal.pone.0042263)

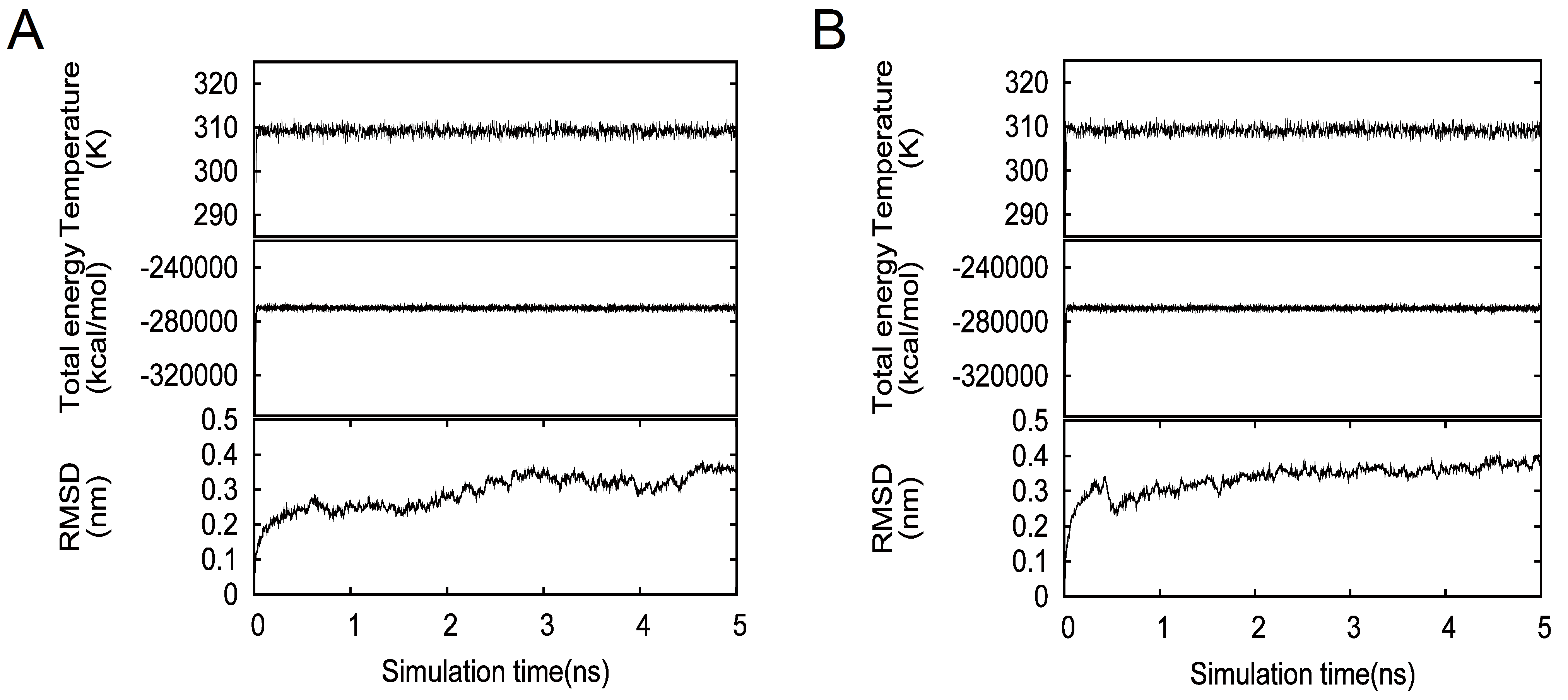

Supplement: Figure S1 — Variation of the temperature, total energy and RMSD of heavy atoms of 6B4/GPIbα complex against simulation time. (A) and (B) express the time-courses of the temperature, total energy and RMSD of heavy atoms of 6B4/GPIbα complex in two independent system equilibrium processes, respectively. The time step is 2 fs. (TIF) [file pone.0042263.s001.tif]

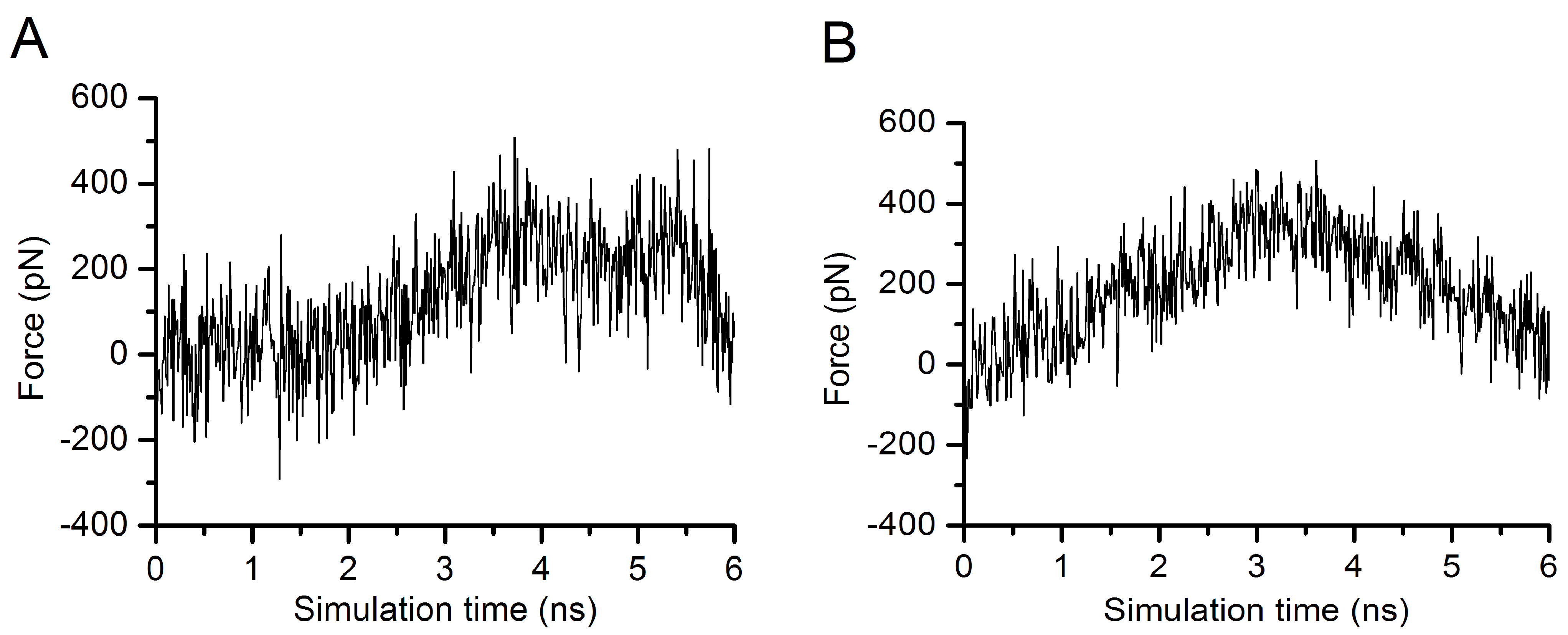

Supplement: Figure S2 — Variation of force on complex under stretching against simulation time. (A) and (B) are the force profiles with two different initial equilibrated complex conformations, respectively, at pulling velocity of 1 nm/ns. The time step is 2 fs, and the data are means of three independent unbinding events. (TIF) [file pone.0042263.s002.tif]

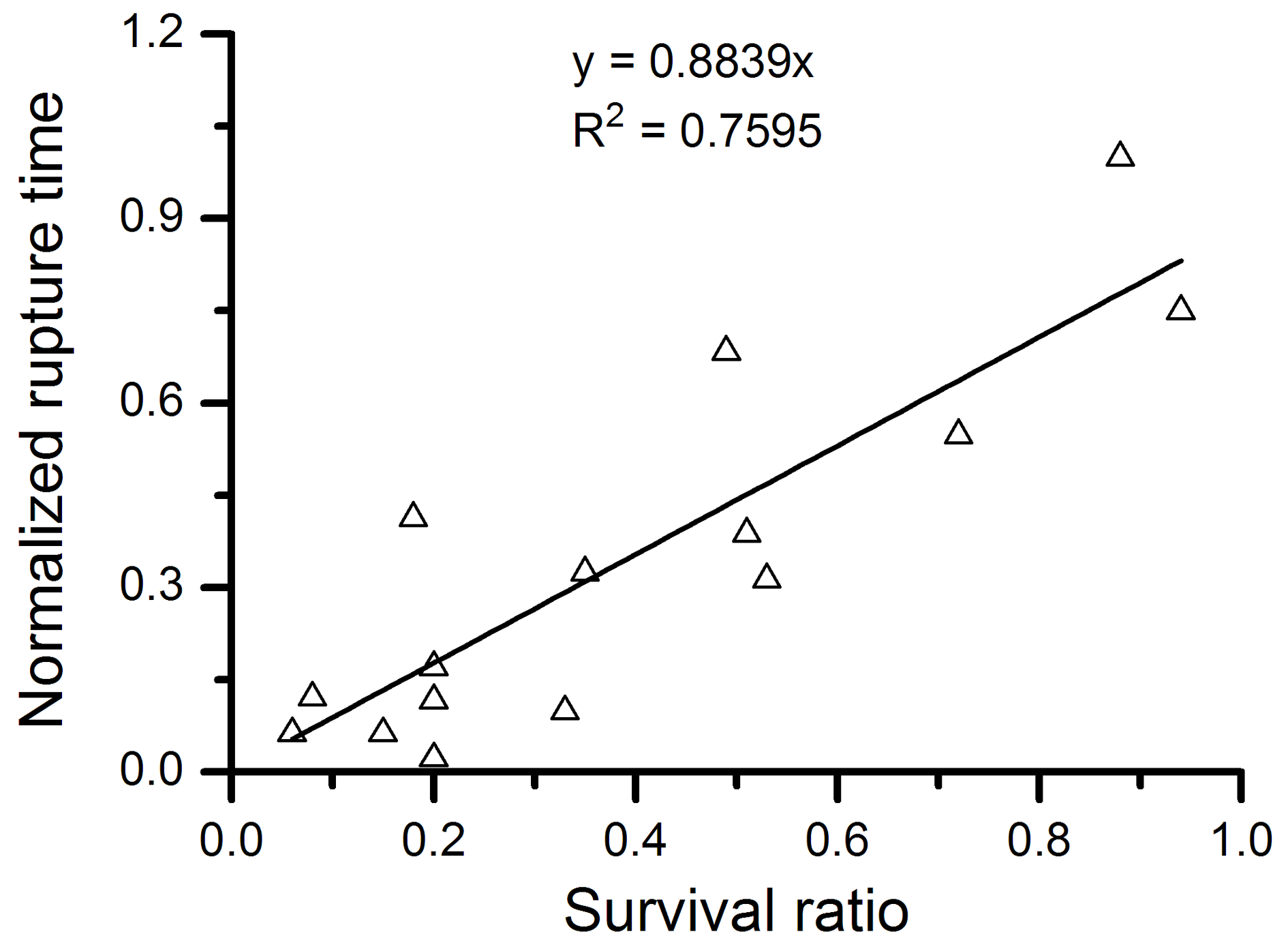

Supplement: Figure S3 — Correlation between normalized rupture time and survival ratio. (TIF) [file pone.0042263.s003.tif]
