# Supplementary material for: Mapping Paratope on Antithrombotic Antibody 6B4 to Epitope on Platelet Glycoprotein Ibalpha via Molecular Dynamic Simulations
Source: PLoS One. 2012 Jul 30;7(7):e42263. doi: 10.1371/journal.pone.0042263 (PMC3408434; doi:10.1371/journal.pone.0042263)
Supplement: Table S1 — Summary of the identified positive and negative residues on 6B4 and GPIbα. (DOC) [file pone.0042263.s005.doc]

**Table S1.** Summary of the identified positive and negative residues on 6B4 and GPIbα.

| No | Residue | Position | Involved Bond(s) | Mean survival ratio | Normalized mean rupture time | HBSI |
| --- | --- | --- | --- | --- | --- | --- |
| 1 | Lys^152^ | GPIbα | 9 | TP | TP | TP |
| 2 | Asp^235^ |  | 1–3 | TP | TP | TP |
| 3 | Asp^175^ |  | 4 | TN | FP | FP |
| 4 | Asp^168^ | 6B4 | 9 | TP | TP | TP |
| 5 | Tyr^166^ |  | 1 | TP | TP | TP |
| 6 | Glu^233^ |  | 2 | TP | FN | TP |
| 7 | Lys^167^ |  | 3–5 | FN | TP | TP |

Where TP and FP express the involved residues being true and false positive, and TN and FN mean the involved residues being true and false negative. With use of the positive criterion score of 0.55, Each of these residues (Table 3 and 4) was assigned either to the positive cluster for its contribution at least on one bond with high stabilization, or to negative cluster in contrast, measuring with the mean survival ratio, the normalized mean rupture time and the HBSI index, respectively (see Materials and Methods). Statistical difference between the two clusters is significant (*p* < 0.01). Here, the mutagenesis experiment data (Table 1) were used to determine whether a positive or negative residue is false or true. Lys^152^ was also regarded as an epitope residue of GPIbα because mutating Lys^152^ to Ala impaired notably the interaction of 6B4 and GPIbα , and Asp^175^ was not for the less effect of mutating Asp^175^ to Ala on binding . Other negative residues were listed in Table S2.
