# Supplementary material for: Mapping Paratope on Antithrombotic Antibody 6B4 to Epitope on Platelet Glycoprotein Ibalpha via Molecular Dynamic Simulations
Source: PLoS One. 2012 Jul 30;7(7):e42263. doi: 10.1371/journal.pone.0042263 (PMC3408434; doi:10.1371/journal.pone.0042263)
Supplement: Table S2 — Unidentified negative residues and their false negative probabilities. (DOC) [file pone.0042263.s006.doc]

**Table S2.** Unidentified negative residues and their false negative probabilities.

| No | Residue | Position | Involved Bond(s) | False negative probability | | |
| --- | --- | --- | --- | --- | --- | --- |
|  |  |  |  | Mean survival ratio | Normalized mean  rupture time | HBSI |
| 1 | Gly^233^ | GPIbα | 7-8 | 0.51 | 0.39 | 0.45 |
| 2 | Gln^232^ |  | 10-15 | 0.53 | 0.31 | 0.42 |
| 3 | Val^234^ |  | 6 | 0.35 | 0.33 | 0.34 |
| 4 | Glu^40^ |  | 18-20 | 0.15 | 0.41 | 0.28 |
| 5 | His^86^ |  | 23 | 0.20 | 0.17 | 0.19 |
| 6 | Glu^181^ |  | 17 | 0.31 | 0.00 | 0.16 |
| 7 | Glu^151^ |  | 5 | 0.08 | 0.12 | 0.10 |
| 8 | Lys^237^ |  | 21-22 | 0.03 |  | 0.02 |
| 9 | Asn^110^ |  | 16 |  | 0.01 | 0.01 |
| 10 | Thr^101^ | 6B4 | 6-8 | 0.51 | 0.39 | 0.45 |
| 11 | Tyr^234^ |  | 10 | 0.53 | 0.31 | 0.42 |
| 12 | Arg^97^ |  | 20 | 0.15 | 0.41 | 0.28 |
| 13 | Ser^100^ |  | 11-12 | 0.20 | 0.19 | 0.19 |
| 14 | Tyr^32^ |  | 23 | 0.20 | 0.17 | 0.19 |
| 15 | Tyr^110^ |  | 18-19 | 0.20 | 0.12 | 0.16 |
| 16 | Ser^163^ |  | 22 | 0.06 | 0.06 | 0.06 |
| 17 | Arg^31^ |  | 15-17 | 0.31 |  | 0.16 |
| 18 | Thr^53^ |  | 13 | 0.05 |  | 0.03 |
| 19 | Leu^164^ |  | 21 | 0.03 |  | 0.02 |

Where the nineteen unidentified residues listed in column 2 were assigned to the negative cluster because their involved bonds of stabilization indices (the mean survival ratio, the normalized mean rupture time and the HBSI index) (see Materials and Methods, Table 3 and 4 as well as Table S1). The residues involved in 14^th^ bond here was missed because it had not been observed from both free and steered MD simulations, the blank spaces in Column 5 and 6 meant the corresponding residues had not been observed from free or steered MD simulations, and these residues were not considered in numbering the corresponding positive test samples. A residues (Column 2) might be involved in one or more bonds (Column 4), its false negative probabilities (column 5, 6 and 7) was measured either to be the mean survival ratio, the normalized mean rupture time or the HBSI value if only one bond was involved, or to be the maxima of the mean survival ratios, the normalized mean rupture times and the HBSI values of involved bonds, respectively. As an example in later case, Gln^232^ contributed to 6 bonds from the 10^th^ to the 15^th^ bond, its false negative probabilities was equal to 0.53 (see cross point of Column 5 and Line 2), the maximum of the mean survival ratios of these six involved bonds.
