# Supplementary material for: Mapping Paratope on Antithrombotic Antibody 6B4 to Epitope on Platelet Glycoprotein Ibalpha via Molecular Dynamic Simulations
Source: PLoS One. 2012 Jul 30;7(7):e42263. doi: 10.1371/journal.pone.0042263 (PMC3408434; doi:10.1371/journal.pone.0042263)
Supplement: Table S3 — The numbers of positive and negative residues derived from three different positive criterions. (DOC) [file pone.0042263.s007.doc]

**Table S3.** The numbers of positive and negative residues derived from three different positive criterions

| Positive criterion | Number of positive and negative residues | | | | | |
| --- | --- | --- | --- | --- | --- | --- |
|  | TP | FP | TN_0_ | FN_0_ | FN_1_ | M |
| Mean survival ratio | 5 | 0 | 1 | 1 | 4.4 | 18 |
| Normalized mean rupture time | 5 | 1 | 0 | 1 | 3.4 | 15 |
| HBSI | 6 | 1 | 0 | 0 | 3.9 | 19 |

Where TP and FP express number of the true and false positive identified-residues, TN_0_ and FN_0_ express the true and false negative identified-residue numbers, respectively, M is the predicted negative residue number, and FN_1_, the possible number of false negative residues in all predicted negative residues of M, were evaluated by Eq. 2. TN and FN, the numbers of all true and false negative residues, were evaluated by TN = TN_0_ +(M-FN_1_), FN= FN_0_ + FN_1_, respectively (see Materials and Methods, Table S1 and S2).
